# Massive Hemoptysis Revealing Rasmussen’s Aneurysm in Active Pulmonary Tuberculosis: A Report of a Fatal Case

**DOI:** 10.7759/cureus.109351

**Published:** 2026-05-21

**Authors:** Mohammed Aharmim, Mohamed Lakhal, Nezha Reguig, Jamal Eddine El bourkadi

**Affiliations:** 1 Department of Pulmonology and Phthisiology, Moulay Youssef Hospital, Rabat, MAR; 2 Department of Respiratory Diseases, Faculty of Medicine and Pharmacy, Centre Hospitalier Universitaire (CHU) Mohammed VI, Mohammed I University, Oujda, MAR; 3 Department of Pulmonology, Faculty of Medicine and Pharmacy, Moulay Youssef Hospital, Mohammed V University, Rabat, MAR; 4 Department of Pulmonology, Moulay Youssef Hospital, Centre Hospitalier Universitaire (CHU) Ibn Sina, Rabat, MAR

**Keywords:** case report, massive hemoptysis, pulmonary artery pseudoaneurysm, pulmonary tuberculosis, rasmussen’s aneurysm

## Abstract

Rasmussen’s aneurysm is a rare but potentially fatal vascular complication of pulmonary tuberculosis and an uncommon cause of massive hemoptysis. A 27-year-old female with no significant past medical history was admitted for a seven-month history of productive cough, intermittent mild hemoptysis, fever, night sweats, and marked asthenia. Chest computed tomography revealed multiple bilateral cavitary lesions and a focal dilatation of a segmental pulmonary artery within a left basal alveolar consolidation, consistent with Rasmussen’s aneurysm. Sputum Xpert *Mycobacterium tuberculosis*/rifampicin (MTB/RIF) assay (Cepheid, Sunnyvale, CA) confirmed drug-susceptible pulmonary tuberculosis. Laboratory testing showed microcytic hypochromic anemia with a hemoglobin level of 7.6 g/dL. The patient received intravenous hemostatic therapy and urgent transfusion of two units of packed red blood cells. Endovascular embolization was planned within 24 hours; however, she died shortly afterward from sudden massive hemoptysis. This case highlights that Rasmussen’s aneurysm may complicate active pulmonary tuberculosis at initial presentation, particularly in the setting of delayed diagnosis, and underscores the need for rapid vascular imaging and urgent multidisciplinary management in any patient with cavitary tuberculosis and hemoptysis.

## Introduction

Tuberculosis remains a major global public health problem and is the leading cause of death from a single infectious agent, ranking among the top 10 causes of mortality worldwide [1]. Although pulmonary tuberculosis primarily affects the lung parenchyma, its complications may also involve the vascular, pleural, and mediastinal compartments.

Hemoptysis is a frequent complication of pulmonary tuberculosis, most commonly originating from the bronchial circulation. However, in rare cases, it may arise from the pulmonary arterial system, particularly in the setting of Rasmussen’s aneurysm, a pseudoaneurysm of a pulmonary artery adjacent to a tuberculous cavity [2,3].

Massive hemoptysis constitutes a life-threatening emergency requiring urgent diagnostic evaluation and immediate therapeutic management, as it may rapidly lead to asphyxia and death [4]. We report a fatal case of Rasmussen’s aneurysm in a 27-year-old female with active pulmonary tuberculosis, highlighting the clinical presentation, radiological findings, and therapeutic challenges associated with this rare but devastating condition.

## Case presentation

A 27-year-old female, employed as an information technology engineer, with no significant past medical history, presented with a seven-month history of productive cough with mucopurulent sputum. The clinical course was marked by intermittent episodes of low-volume hemoptysis associated with constitutional symptoms, including weight loss, marked asthenia, fever, and night sweats.

**Figure 1:**
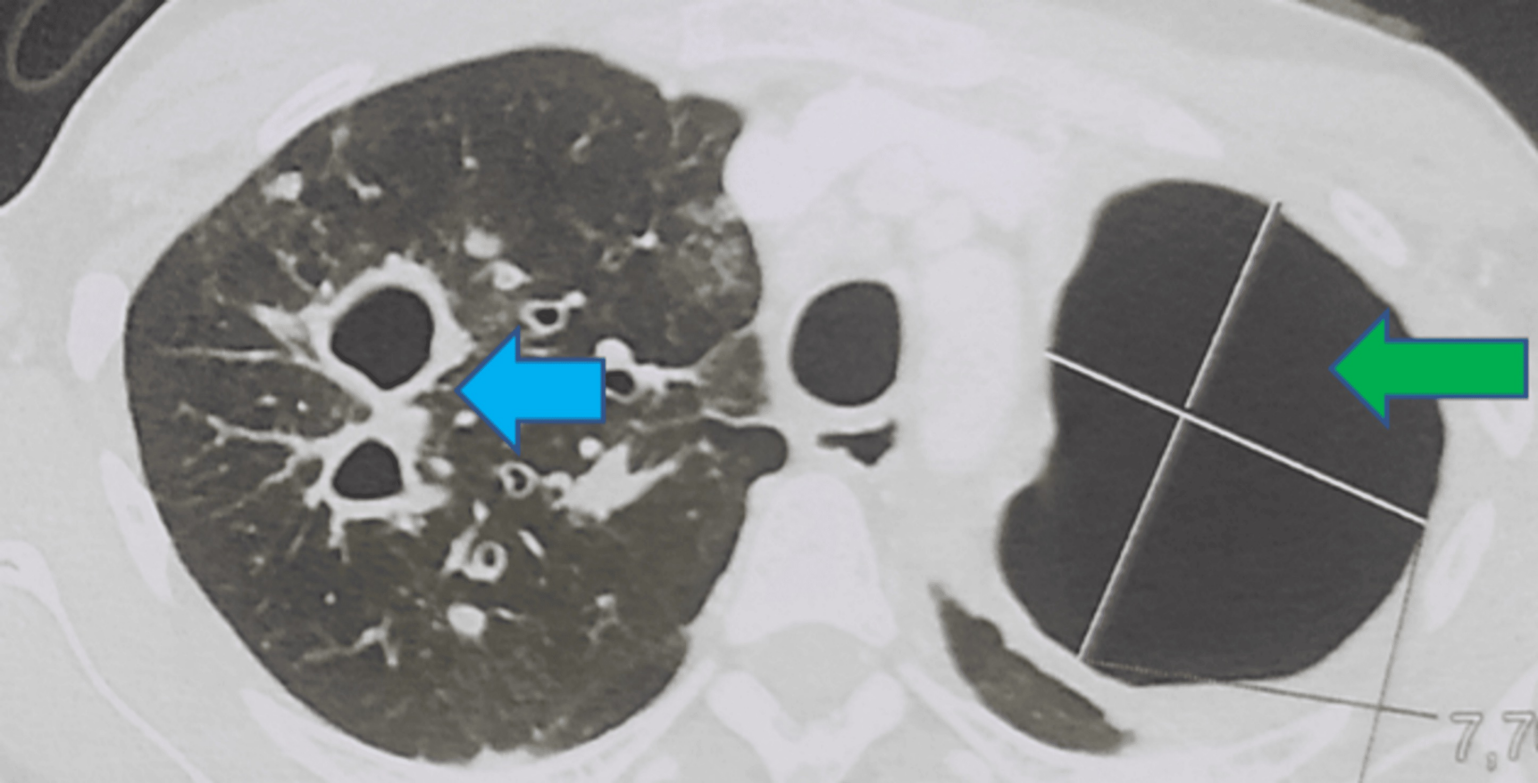
Large cavitary lesion in the left upper lobe measuring 77 × 66 mm (green arrow), associated with cavitary changes and bronchiectasis in the right upper lobe (blue arrow).

**Figure 2:**
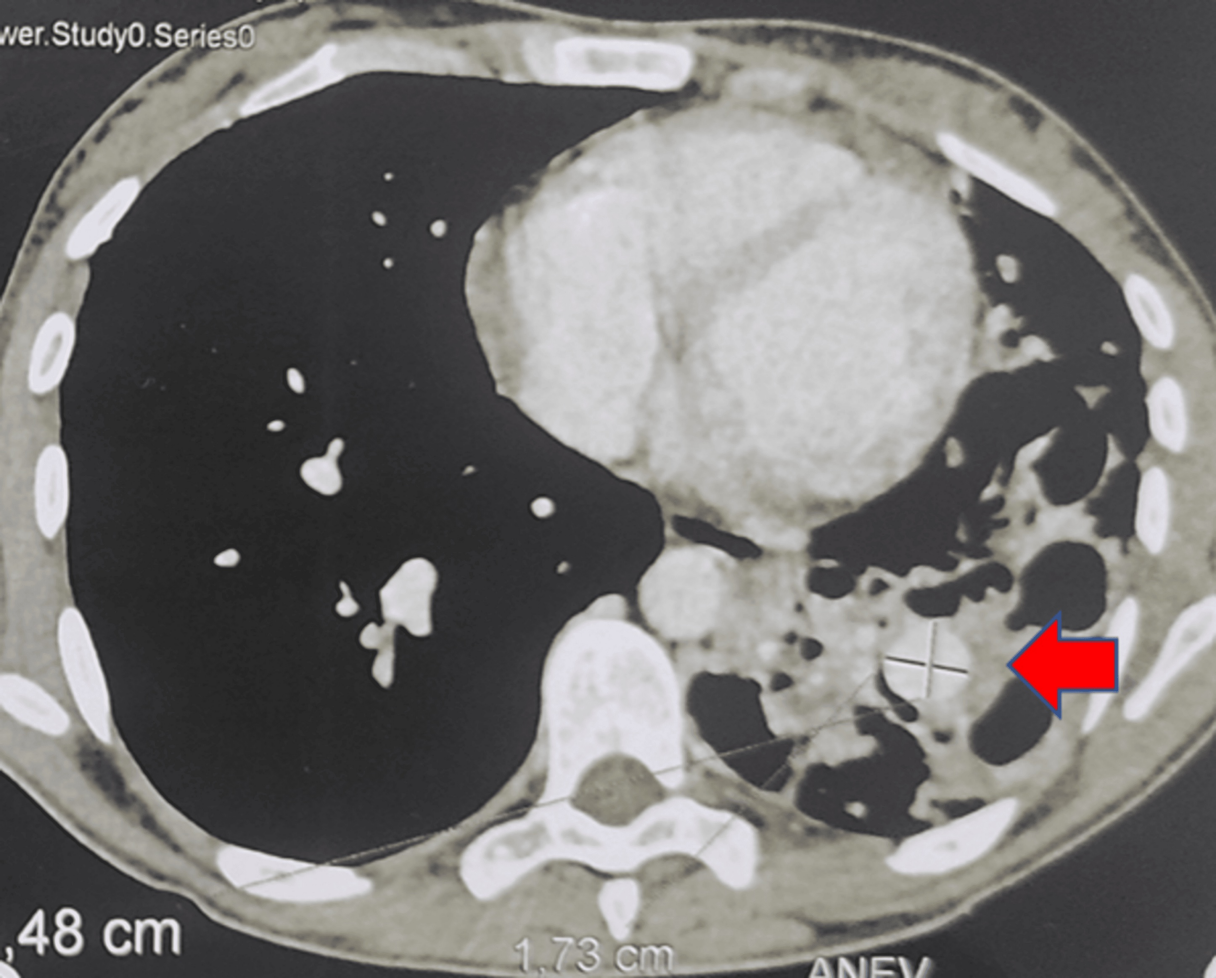
Rasmussen’s aneurysm measuring 17.3 × 14.8 mm within a left basal pulmonary consolidation (red arrow).

On admission, she was conscious and hemodynamically stable, with preserved oxygen saturation at rest, normal blood pressure, and sinus tachycardia at 110 beats/minute. Chest computed tomography revealed multiple large, thick-walled cavitary lesions involving both left pulmonary lobes, the right upper lobe, and the apical segment of the right lower lobe. The largest cavity was located in the left upper lobe and measured 77 × 66 mm (Figure 1). Computed tomography also demonstrated a left basal alveolar consolidation containing a focal dilatation of a segmental pulmonary artery measuring 17.3 × 14.8 mm, consistent with Rasmussen’s aneurysm (Figure 2).

Sputum Xpert *Mycobacterium tuberculosis*/rifampicin (MTB/RIF) assay (Cepheid, Sunnyvale, CA) confirmed drug-susceptible pulmonary tuberculosis. Laboratory evaluation revealed microcytic hypochromic anemia with a hemoglobin level of 7.6 g/dL, while renal and liver function tests were within normal limits. The patient received intravenous hemostatic therapy with etamsylate and tranexamic acid, along with an urgent transfusion of two units of packed red blood cells. Endovascular embolization was scheduled within 24 hours; however, the patient died shortly afterward following a fulminant episode of massive hemoptysis.

## Discussion

Rasmussen’s aneurysm is an uncommon but highly lethal vascular complication of cavitary pulmonary tuberculosis. It results from progressive weakening of the pulmonary arterial wall adjacent to a tuberculous cavity, leading to pseudoaneurysm formation and potential rupture [5,6]. Although hemoptysis is common in tuberculosis, bleeding of pulmonary arterial origin remains significantly less frequent than that arising from the bronchial circulation [2].

One of the most notable features of this case is the timing of diagnosis. In most reported cases, Rasmussen’s aneurysm is identified in patients with post-tuberculous cavitary sequelae or advanced disease [6,7]. In contrast, our patient was diagnosed during the active phase of tuberculosis, suggesting a significant diagnostic delay that allowed progression to extensive cavitation and vascular involvement. This observation highlights that Rasmussen’s aneurysm should not be regarded solely as a late complication but may also occur during active disease.

Another important aspect is the social context. While tuberculosis is traditionally associated with poverty and socioeconomic deprivation, diagnostic delays may occur across all social strata. Contributing factors include psychosocial vulnerability, stigma, and barriers to healthcare access [8]. In this case, despite a favorable socioeconomic background, delayed presentation was likely influenced by family conflict and emotional stress, underscoring the broader role of social determinants of health in disease progression.

From a diagnostic perspective, contrast-enhanced chest computed tomography, particularly computed tomography angiography, plays a crucial role. It enables accurate identification of the bleeding source and differentiation between pulmonary and bronchial arterial origins, which is essential for appropriate management [2,4].

Regarding treatment, endovascular embolization is currently considered the first-line therapy when available. It allows rapid and minimally invasive control of bleeding, commonly using coil embolization techniques [3,9]. Depending on the vascular anatomy, embolization may target the pulmonary artery, bronchial arteries, or both. Surgical intervention remains a rescue option when embolization fails, is not feasible, or in cases of localized destructive pulmonary disease [10].

The fatal outcome in this case, despite planned embolization, underscores the extreme time sensitivity of Rasmussen’s aneurysm once massive hemoptysis occurs. It highlights the need for rapid diagnosis, immediate access to interventional radiology, and close multidisciplinary collaboration among pulmonologists, radiologists, intensivists, and thoracic surgeons.

## Conclusions

Rasmussen’s aneurysm is a rare but devastating complication of pulmonary tuberculosis that may be diagnosed during active disease and not only in post-tuberculous sequelae. In patients with cavitary pulmonary tuberculosis and hemoptysis, early CT angiographic assessment is essential to identify this potentially fatal vascular lesion. Prompt multidisciplinary management and timely endovascular intervention may be lifesaving, as fatal hemorrhage can occur before definitive treatment is performed.

## References

[1] Ayalew YE, Yehualashet FA, Bogale WA, Gobeza MB (2020). Delay for tuberculosis treatment and its predictors among adult tuberculosis patients at Debremarkos Town public health facilities, North West Ethiopia. Tuberc Res Treat.

[2] Khalil A, Parrot A, Nedelcu C, Fartoukh M, Marsault C, Carette MF (2008). Severe hemoptysis of pulmonary arterial origin: signs and role of multidetector row CT angiography. Chest.

[3] Picard C, Parrot A, Boussaud V, Lavolé A, Saidi F, Mayaud C, Carette MF (2003). Massive hemoptysis due to Rasmussen aneurysm: detection with helicoidal CT angiography and successful steel coil embolization. Intensive Care Med.

[4] Park HS, Chamarthy MR, Lamus D, Saboo SS, Sutphin PD, Kalva SP (2018). Pulmonary artery aneurysms: diagnosis & endovascular therapy. Cardiovasc Diagn Ther.

[5] Mahmoud N, Moussa C, Attia M (2022). Rasmussen aneurysm: a forgotten complication of tuberculosis in the COVID-19 era. Respir Med Case Rep.

[6] Marak JR, Kumar T, Gara H, Dwivedi S (2023). Rasmussen aneurysm: case series of a rare complication of pulmonary tuberculosis. Respir Med Case Rep.

[7] Seedat UF, Seedat F (2018). Post-primary pulmonary TB haemoptysis - when there is more than meets the eye. Respir Med Case Rep.

[8] Bonadonna LV, Saunders MJ, Zegarra R, Evans C, Alegria-Flores K, Guio H (2017). Why wait? The social determinants underlying tuberculosis diagnostic delay. PLoS One.

[9] Rajamannar KV, Kilaru H, Aravelly S, Gudipati AR, Kilaru SC (2017). Massive hemoptysis from Rasmussen's aneurysm in active pulmonary tuberculosis; a case report of successful treatment with bronchial artery embolization. Respir Med Case Rep.

[10] Rengan SS, Rani P, Rathi RK, Joseph HT, Mohan A, Bal S (2025). Surgical repair of Rasmussen aneurysm of left pulmonary artery-case report. Indian J Thorac Cardiovasc Surg.

